# Coordination between midcingulate cortex and retrosplenial cortex in pain regulation

**DOI:** 10.3389/fnmol.2024.1405532

**Published:** 2024-08-06

**Authors:** Yunya Qiu, Yan-Na Lian, Cheng Wu, Li Liu, Chen Zhang, Xiang-Yao Li

**Affiliations:** ^1^NHC and CAMS Key Laboratory of Medical Neurobiology, MOE Frontier Science Center for Brain, Research and Brain-Machine Integration, School of Brain Science and Brain Medicine, Zhejiang University, Hangzhou, China; ^2^Department of Psychiatry, The Fourth Affiliated Hospital of Zhejiang University School of Medicine and International Institutes of Medicine, Zhejiang University School of Medicine, Yiwu, China; ^3^Core Facilities of the School of Medicine, Zhejiang University, Hangzhou, China; ^4^State Key Laboratory of Neurology and Oncology Drug Development, Nanjing, China; ^5^Beijing Key Laboratory of Neural Regeneration and Repair & Beijing Laboratory of Oral Health, School of Basic Medical Sciences, Capital Medical University, Beijing, China; ^6^Chinese Institute for Brain Research, Beijing, China

**Keywords:** chemogenetic approach, pain regulation, aversion, neuropathic pain, MCC, RSC

## Abstract

**Introduction:**

The cingulate cortex, with its subregions ACC, MCC, and RSC, is key in pain processing. However, the detailed interactions among these regions in modulating pain sensation have remained unclear.

**Methods:**

In this study, chemogenetic tools were employed to selectively activate or inhibit neuronal activity in the MCC and RSC of rodents to elucidate their roles in pain regulation.Results: Our results showed that chemogenetic activation in both the RSC and MCC heightened pain sensitivity. Suppression of MCC activity disrupted the RSC’s regulation of both mechanical and thermal pain, while RSC inhibition specifically affected the MCC’s regulation of thermal pain.

**Discussion:**

The findings indicate a complex interplay between the MCC and RSC, with the MCC potentially governing the RSC’s pain regulatory mechanisms. The RSC, in turn, is crucial for the MCC’s control over thermal sensation, revealing a collaborative mechanism in pain processing.

**Conclusion:**

This study provides evidence for the MCC and RSC’s collaborative roles in pain regulation, highlighting the importance of their interactions for thermal and mechanical pain sensitivity. Understanding these mechanisms could aid in developing targeted therapies for pain disorders.

## 1 Introduction

Pain is the unpleasant sensory and emotional experience associated with actual or potential tissue damage (Raja et al., 2020). Pain perception typically serves as a protective warning signal under normal circumstances. When pain persists for over 3 months, it is classified as chronic pain, a condition that significantly contributes to disability and distress, impacting more than 20% of the global population (Lindsay et al., 2021). Research on both animals and humans has identified key neocortical areas involved explicitly in pain based on distinct circuit connections and cellular ensembles (Vogt, 2005; Shackman et al., 2011; Tan and Kuner, 2021).

The cingulate cortex has a critical role in emotion, action, memory, and pain (Peyron et al., 2019; Vogt, 2019; Benarroch, 2020). Structural, circuitry, and functional imaging studies have delineated the cingulate cortex into four subregions in humans, including the anterior cingulate cortex (ACC), midcingulate cortex (MCC), posterior cingulate cortex (PCC), and retrosplenial cortices (RSC) (Vogt et al., 1996; Vogt, 2005; Palomero-Gallagher et al., 2009). The ACC’s role in regulating acute and chronic pain has been broadly investigated (Bliss et al., 2016; Tan and Kuner, 2021). Recent research has also highlighted specific roles of the MCC (Tan et al., 2017; Hu et al., 2019; Hong et al., 2023) and RSC (Barrière et al., 2019; Wang et al., 2023) in pain regulation. The neuronal activities within the MCC are integral to the multisensory orientation of the head and body in space, owing to its projections to the spinal cord (Vogt, 2016). On the other hand, the RSC plays a prominent role in visuospatial orientation and memory, facilitated by its reciprocal connections with the anterior thalamic nuclei and the hippocampal formation (Kwapis et al., 2015; Mitchell et al., 2018; Balcerek et al., 2021). These distinct functions underscore the differing roles of the MCC and RSC in pain regulation. Neurons within both the MCC and RSC are activated by the visceral nociceptive and cutaneous nociceptive stimuli (Sikes et al., 2008). Pain-related information is transmitted from the anteromedial thalamus nucleus to the MCC (Hong et al., 2023), which regulates sensory hypersensitivity without causing adverse effects through the afferent MCC-posterior insula pathway (Tan et al., 2017). Additionally, projections from the MCC to GABAergic neurons in the zona incerta mediate its pain-inhibitory function (Hu et al., 2019). Conversely, the RSC primarily contributes to spatial navigation, long-term memory storage, and regulation (Vann et al., 2009; Kwapis et al., 2015; Balcerek et al., 2021). Previous studies have demonstrated the involvement of the role of the RSC in formalin-induced place aversion (Lei et al., 2004), and brain imaging studies have revealed widespread structural and functional changes in the RSC following nerve injury. Furthermore, transcriptomic analyses using single-cell/nucleus RNA sequencing have identified imbalanced excitatory/inhibitory synaptic transmissions, implicated in both sensory hypersensitivity and place aversion after peripheral nerve injury (Wang et al., 2023). These findings suggest that MCC and RSC may have different roles in pain regulation. However, the specific modulations of the MCC and RSC during pain regulation remain to be fully elucidated. In the present study, we sought to assess the interdependence of the MCC and RSC in pain regulation by employing a combination of different methodological approaches.

## 2 Method and materials

### 2.1 Experimental animals

This study utilized adult male C57B L/6 mice (8 weeks, weight: 20–35 g). The mice were housed in groups of four or five per cage under constant environmental conditions, including a room temperature of 21 ± 1°C, relative humidity of 60 ± 5%, and a regular light/dark schedule (light from 7 am–7 pm). Food and water were provided *ad libitum*. The mice were allowed to acclimate to the laboratory conditions for approximately 1 week before behavioral tests and to the testing environment for at least 15 min prior to the experiment. The Zhejiang University Animal Care and Use Committee approved all mouse protocols.

### 2.2 Stereotaxic virus injection

AAV9-CaMKIIα-mCherry (6.34 × 10^13^ vg mL^−1^, 130 nL/unilateral) and rAAV2/1-CaMKIIα-mCherry (5.56 × 10^13^ vg mL^−1^, 130 nL/unilateral) were obtained from Vigene Biosciences (Shandong, China). AAV9-CamkIIα-ChR2-EGFP (1.04 × 10^13^ vg mL^−1^, 130 nL/unilateral), rAAV2-retro-GFP (9.97 × 10^12^ vg mL^−1^, 130 nL/unilateral), AAV9-CaMKIIα-hM3Dq-mCherry (7.9 × 10^12^ vg mL^−1^, 130 nL/unilateral) and AAV9-CaMKIIα-hM4Di-GFP (1.24 × 10^13^ vg mL^−1^, 130 nL/unilateral) were obtained from OBiO Technology Corp., Ltd. (Shanghai, China). Stereotaxic injections of AAVs were performed and adopted (Tian et al., 2018).

In brief, mice were anesthetized using isoflurane (induction 4%, maintenance 1%). The scalp was shaved and then cleaned with iodine (Triadine) and alcohol. The mouse’s head was secured in a stereotaxic adapter mounted on a stereotaxic frame (KOPF model 962). Lubricant (artificial tears) was applied to the eyes to prevent dryness. A surgical incision was made on the scalp to expose the skull surface. Two small holes were drilled above the target brain regions, either the MCC (AP: −0.35 mm, ML: ±0.25 mm, DV: −1.25 mm) or RSC (AP: −1.80 mm, ML: ±0.25 mm, DV: −1.15 mm). The virus was infused at a rate of 20 nL/min. Following the infusion, the needle was left at the injection site for 10 min and then slowly withdrawn. The total volume of the infused virus was based on its titer.

### 2.3 Behavioral tests

#### 2.3.1 Mechanical allodynia test

The von Frey behavioral assay was performed according to the up-down algorithm described by Chaplan et al. (1994). To assess the animals’ responses to mechanical stimuli, they were positioned on an elevated mesh grid and enclosed in a plastic box for confinement. Calibrated von Frey filaments were applied to the center of the plantar surface of each paw until the filaments were bowed. The paw’s prompt withdrawal or flinching was recorded as a positive response, indicating sensitivity to the stimulus. Any lifting of the paw as part of regular locomotor activity was disregarded. In the absence of a response, the next filament with increased force was applied. Conversely, the subsequent filament with reduced force was used after a response. The level of tactile stimulation eliciting a 50% likelihood of withdrawal was determined and denoted as the paw withdrawal threshold (PWT).

#### 2.3.2 Hargreaves test

The Hargreaves test was adapted from a behavioral paradigm reported by Hargreaves et al. (1988). Briefly, the mice were acclimated to the testing environment for 2 h and familiarized with the test arena 3 days before the experiment. The light intensity was set at 16%, with a cut-off time of 20 s. During the test, a radiant heat source was positioned beneath the animal, targeting the plantar surface of the hind paw. The time taken for the mouse to withdraw from the heat stimulus was recorded as the withdrawal latency. Each mouse underwent four repeated tests with the same paw.

### 2.4 Conditioned place aversion

A conditioned place aversion (CPA) test was conducted using the method outlined by a previous study (Wang et al., 2017, 2023). Briefly, the mice underwent a preconditioning phase over the first 2 days, during which they freely explored the chambers for 15 min each day. On the third day, the mice’s activities were recorded for 15 min, and the time spent in each chamber was analyzed. The following day, the mice received an injection of the control solution and were paired with a randomly selected chamber for 30 min in the morning. Four hours later, they received the appropriate drug treatment and were paired with other chambers for 30 min in the afternoon. After a 20 h interval from the afternoon pairing, the mice were placed in the box with access to all chambers, and their behavior was recorded for 15 min to evaluate chamber preference. The different time in each chamber compared to that recorded on the third day was calculated.

### 2.5 Whole-cell patch-clamp recording

Coronal brain slices (300 μm) at the level of the MCC or RSC from mice were prepared. Then, slices were transferred to a submerged recovery chamber with oxygenated (95% O_2_ and 5% CO_2_) artificial cerebrospinal fluid (ACSF) containing (in mM) 124 NaCl, 2.5 KCl, 2 CaCl_2_, 1 MgSO_4_, 25 NaHCO_3_, 1 NaH_2_PO_4_, and 10 glucose at room temperature for at least 1 hour. Experiments were performed in a recording chamber on the stage of a microscope equipped with infrared differential interference contrast optics for visualization.

To confirm the efficacy of GFP-mediated activation, neurons labeled GFP were visualized and stimulated with a laser (Lumen Dynamics, Canada) using 10 Hz stimulation protocols with a 1 ms pulse. Evoked excitatory postsynaptic currents (eEPSCs) were recorded from neurons of the MCC or RSC using a MultiClamp 700B amplifier and Digidata 1550A. The recording micropipettes (3–5 MΩ) were filled with a solution containing (in mM) 115 CsMeSO_4_, 20 CsCl, 10 HEPES, 2.5 MgCl_2_, 4 Na-ATP, 0.4 Na_2_-GTP, 10 Na_2_phosphocreatine, 0.6 EGTA, 5 QX-314 (adjusted to pH 7.3–7.4 with CsOH, 290–300 mOsm). AMPAR-mediated EPSCs were recorded under a voltage-clamp model and the membrane potentials were held at −70 mV. To confirm the involvement of AMPARs, CNQX (20 uM) was used to block the EPSCs (Jiang et al., 2017).

To confirm the efficacy of chemogenetic activation or inhibition, CNO (10 uM) was bath-applied. The internal solution contained (in mM) 124 K-gluconate, 5 NaCl, 1 MgCl_2_, 0.2 EGTA, 10 HEPES, 2 MgATP, 0.1 Na_3_GTP, and 10 phosphocreatine disodium (adjusted to pH 7.3–7.4 with KOH, 290–300 mOsm). The initial access resistance was 15–30 MΩ and was monitored throughout experiments. Data were discarded if the access resistance changed >15% during an experiment. Data were filtered at 1 kHz and digitized at 10 kHz (Li et al., 2010, 2014).

### 2.6 Data analysis

Data analysis and graph plotting were performed using GraphPad Prism 8.0 software. Offline analysis of whole-cell patch-clamp data was performed using Clampfit 10. 6. Statistical comparisons were conducted using appropriate tests such as student’s *t-*test, one-way ANOVA, or two-way ANOVA, followed by *post hoc* comparisons such as Tukey’s, Bonferroni, or Šídák’s test. All data were presented as mean ± standard error of the mean (SEM). A significance threshold of *p* < 0.05 was used to determine statistical significance in all cases.

## 3 Results

### 3.1 Manipulation of the neuronal activities in the RSC via a chemogenetic approach

**Figure 1:**
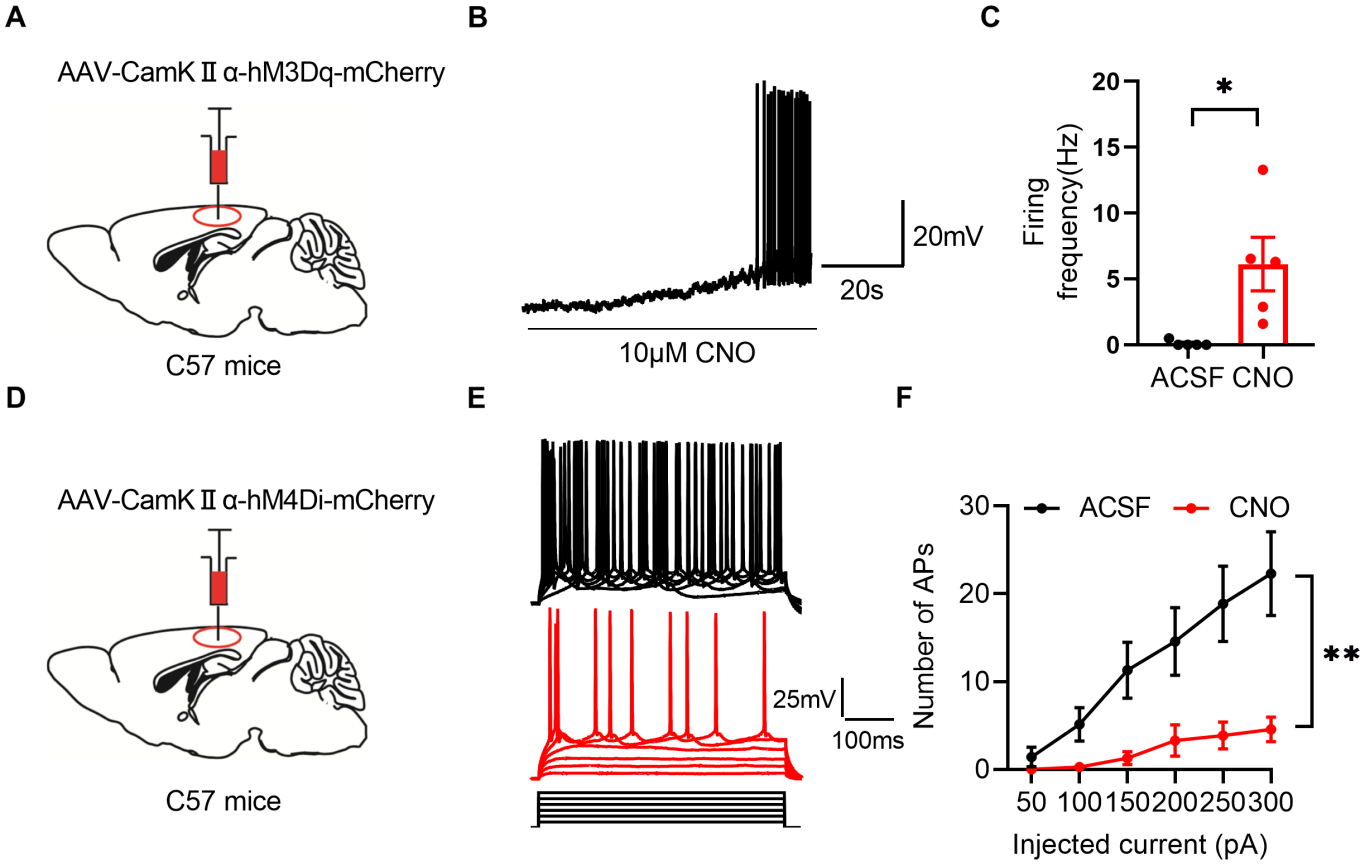
Chemogenetic manipulation of neuron activation or inhibition. **(A)** Illustration of viral delivery of AAV-CamKIIα-hM3Dq-mCherry into the RSC of mice. **(B)** A representative trace showing chemogenetic activation of RSC neurons with AAV-CamKIIα-hM3Dq-mCherry. **(C)** Summary data for the firing frequency of neurons activated by CNO (paired *t*-test, two-tailed, *t* = 2.97, df = 4, *p* = 0.04, 5 neurons from 3 mice). **(D)** Illustration of viral delivery of AAV-CamKIIα-hM4Di-mCherry into the RSCof mice. **(E)** Representative traces showing chemogenetic inhibition of RSC neurons with AAV-CamKIIα-hM4Di-mCherry. **(F)** Quantification of the number of action potentials (APs) fired by RSC neurons inhibited by CNO. (Two-way ANOVA, Interaction, *F*_(5, 60)_ = 9.21, *p* < 0.0001, ACSF vs. CNO: *F*_(1, 12)_ = 10.29, *p* < 0.01, 7 neurons from 3 mice).

To investigate the involvement of RSC and MCC in pain regulation, we employed the chemogentic approach to manipulate the neuronal activities in the RSC or MCC. We first used adeno-associated virus 9 (AAV9) to express the human M3 muscarinic designer receptor (hM3Dq) (AAV9-CaMKIIα-hM3Dq-mCherry) or the human M4 muscarinic designer receptor (AAV9-CaMKIIα-hM4Di-mCherry) into the RSC, and three weeks after virus injection, we performed the whole-cell patch-clamp recording experiments on the mCherry expressing neurons, as showed in Figure 1, bath application of clozapine-N-oxide (CNO, 10 μM) significantly increased (Figures 1A–C) or decreased (Figures 1D–F) the firing frequency of the action potentials on the hM3Dq or hM4Di expressing groups, respectively. These results confirmed the effects of CNO on the activities of the virus-expressing neurons.

### 3.2 Elevation of the RSC’s neuronal activities chemogenetically sensitized the pain sensation and induced place aversion

**Figure 2:**
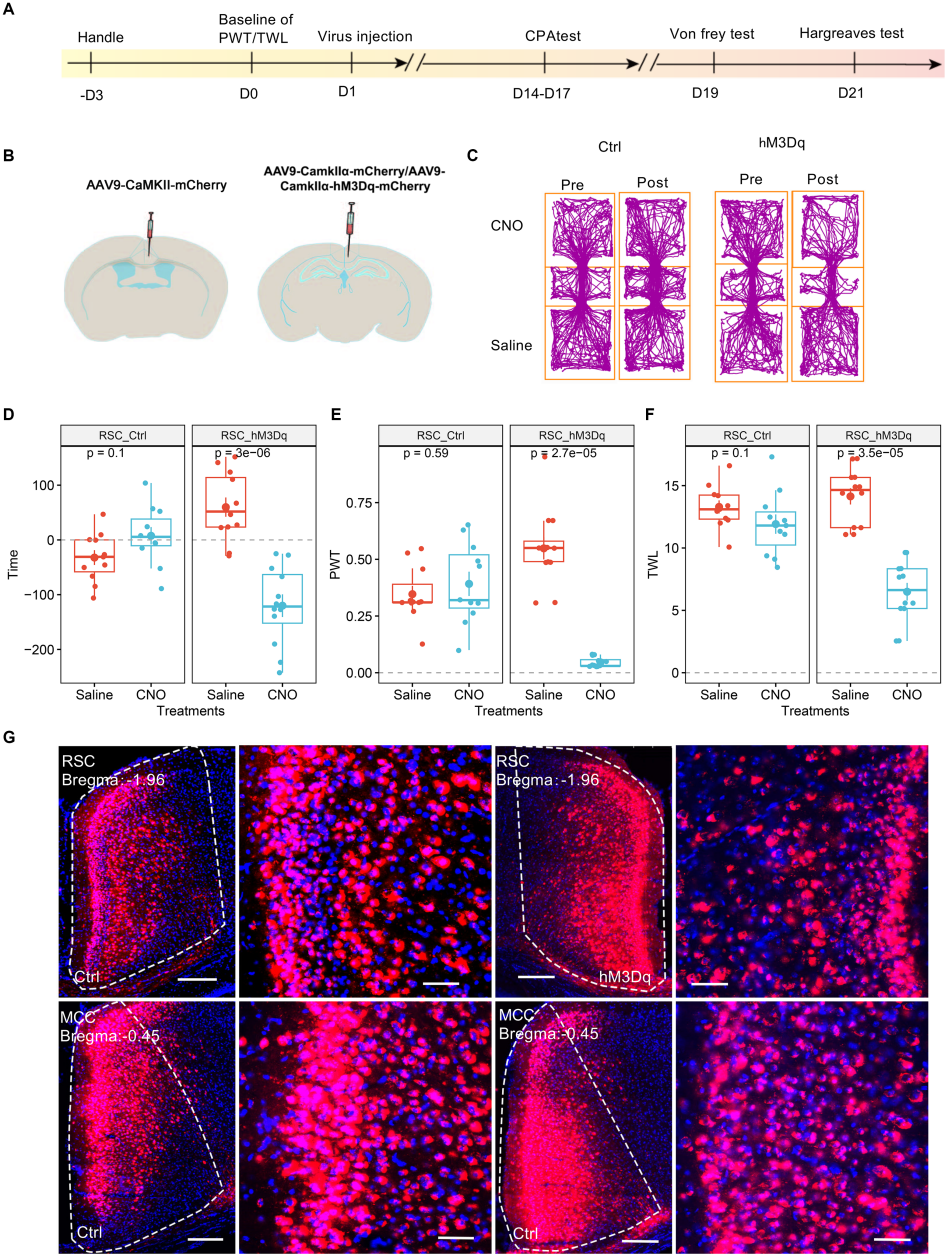
Increasing the activation of RSC neurons decreases PWT, shortens TWL, and induces conditioned place aversion. **(A)** The cartoon diagram illustrates the experimental procedure of applying chemogenetics to elevate neuronal activity in the RSC and to perform behavioral assessments in the current research. **(B)** Schematic diagram of virus injection in mouse brain area. **(C)** Representative traveling traces of the control (Ctrl) and hM3Dq group of mice in the conditioned place aversion test. **(D)** Place aversion induced by CNO application in the hM3Dq_RSC group. (Two-way ANOVA, interaction, *F*_(1, 21)_ = 23.18, *p* < 0.0001, Saline vs. CNO: *F*_(1, 21)_ = 9.43, *p* < 0.01; Ctrl vs. hM3Dq: *F*_(1, 21)_ = 3.i95, *p* = 0.06; Tukey’s multiple comparisons test, hM3Dq: Saline vs. CNO, *p* < 0.0001, Ctrl: *n* = 11, hM3Dq: *n* = 12). **(E)** The application of CNO decreased the paw withdrawal thresholds (PWT) in hM3Dq-expressing mice. (Two-way ANOVA, Interaction, *F*_(1, 21)_ = 82.45, *p* < 0.001, Saline vs. CNO: *F*_(1, 21)_ = 48.33, *p* < 0.0001, Ctrl vs. hM3Dq: *F*_(1, 21)_ = 13.48, *p* < 0.01; Tukey’s multiple comparisons test, hM3Dq: Saline vs. CNO, *p* < 0.0001, Ctrl: *n* = 11, hM3Dq: *n* = 12). **(F)** The application of CNO extended the thermal withdrawal latency (TWL) in hM3Dq-expressing mice. (Two-way ANOVA, Interaction, *F*_(1, 21)_ = 33.13, *p* < 0.0001; Saline vs. CNO, *F*_(1, 21)_ = 84.75, *p* < 0.0001; Ctrl vs. hM3Dq: *F*_(1, 21)_ = 4.84, *p* < 0.05; Tukey’s multiple comparisons test, hM3Dq: Saline vs. CNO, *p* < 0.0001, Ctrl: *n* = 11, hM3Dq: *n* = 12). **(G)** Representative images showing expression of Ctrl or hM3Dq-mCherry in the RSC.

We investigated the potential involvement of splenial neurons in pain regulation. Consistent with a previous study (Wang et al., 2023), we injected AAV9-CaMKIIα-hM3Dq-mCherry specifically in the RSC region. As a control, AAV9-CaMKIIα-mCherry was used (Figures 2A,B). Subsequently, we administered CNO (1 mg/kg, i.p., intraperitoneal injection) to activate the RSC neurons. In conditioned place aversion experiments, the application of CNO significantly induced place aversion in mice expressing the hM3Dq receptor (Figures 2C,D). Behaviorally, CNO administration led to a decrease in the PWT (Figure 2E) and a reduction in thermal withdrawal latency (TWL) in mice expressing the hM3Dq receptor (Figure 2F). The expression of the virus was confirmed using fluorescence imaging (Figure 2G). These results are consistent with our previous study (Wang et al., 2023), indicating that increased activation of the RSC neurons enhances mechanical and thermal sensitivity, leading to aversive behavior.

### 3.3 Boosting the MCC’s neurons’ activities chemogenetically sensitized the pain sensation

**Figure 3:**
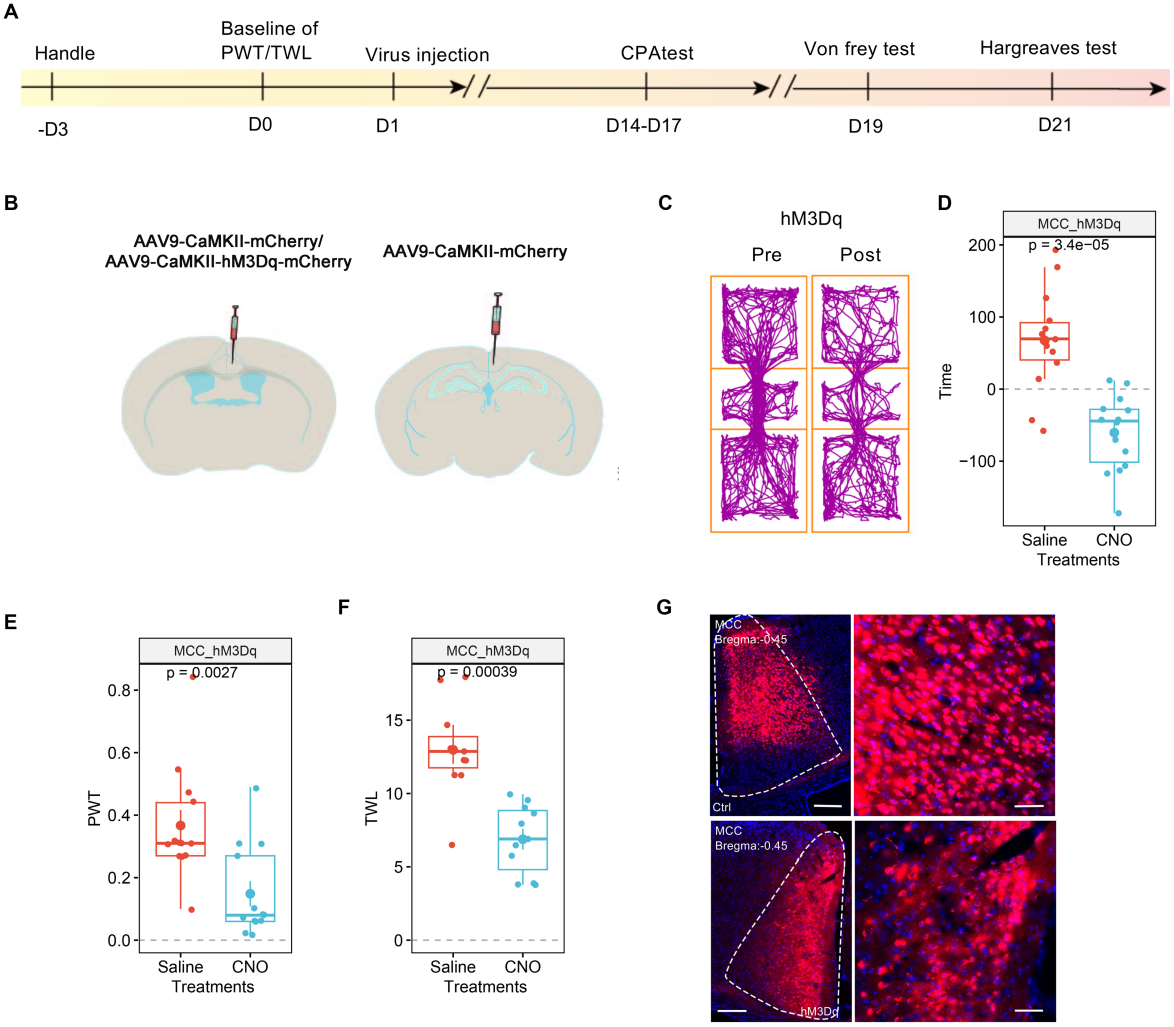
Increasing the activation of MCC neurons decreases PWT and shortens TWL. **(A)** The cartoon diagram illustrates the experimental procedure of applying chemogenetics to elevate neuronal activity in the MCC and to perform behavioral assessments in the current research. **(B)** Schematic diagram of virus injection in the MCC and RSC. **(C)** Representative traveling traces of the Ctrl and hM3Dq group of mice in the conditioned place aversion test. **(D)** The CNO application in the hM3Dq (MCC) group induced place aversion compared to the RSC_Ctrl_MCC_Ctrl in Figure 2D. (Two-way ANOVA, Interaction, *F*_(1, 23)_ = 16.46, *p* < 0.0005, Saline vs. CNO: *F*_(1, 23)_ = 4.55, *p* < 0.05, Ctrl vs. hM3Dq: *F*_(1, 23)_ = 2.75, *p* = 0.11; Tukey’s multiple comparisons test, hM3Dq: Saline vs. CNO, *p* < 0.001, Ctrl: *n* = 11, hM3Dq: *n* = 14). **(E)** The application of CNO decreased the PWT in hM3Dq-expressing mice compared to the RSC_Ctrl_MCC_Ctrl in Figure 2E. (Two-way ANOVA, Interaction, *F*_(1, 22)_ = 11.31, *p* < 0.01, Saline vs. CNO: *F*_(1, 22)_ = 5.98, *p* < 0.05, Ctrl vs. hM3Dq: *F*_(1, 22)_ = 4.13, *p* > 0.05; Tukey’s multiple comparisons test, hM3Dq: Saline vs. CNO, *p* < 0.01, Ctrl: *n* = 11, hM3Dq: *n* = 13). **(F)** The application of CNO extended the TWL in hM3Dq-expressing mice compared to the RSC_Ctrl_MCC_Ctrl in Figure 2F. (Two-way ANOVA, Interaction, *F*_(1, 20)_ = 9.14, *p* < 0.01, Saline vs. CNO: *F*_(1, 20)_ = 39.23, *p* < 0.001, Ctrl vs. hM3Dq: *F*_(1, 20)_ = 5.41, *p* < 0.05; Tukey’s multiple comparisons test, hM3Dq: Saline vs. CNO, *p* < 0.001, Ctrl: *n* = 11, hM3Dq: *n* = 11), ^***^indicates *p* < 0.001. **(G)** Representative images showing expression of control or hM3Dq-mCherry in the MCC.

We then investigated the involvement of MCC in pain regulation. Similarly, we expressed the AAV9-CaMKIIα-hM3Dq-mCherry, with AAV9-CaMKIIα-mCherry as the control, in the MCC (Figures 3A,B). After achieving stable expression, we systemically administered CNO (1 mg/kg, i.p.) to activate the MCC neurons. Similar to the results of the hM3Dq expression in the RSC, the application of CNO did induce place aversion in mice expressing the hM3Dq receptor in the MCC (Figures 3C,D). Furthermore, CNO administration decreased the PWT (Figure 3E) and shortened the TWL (Figure 3F) in mice expressing the hM3Dq receptor. The expression of the virus was further confirmed through fluorescence imaging (Figure 3G). Therefore, increasing the activation of the MCC neurons sensitized both mechanical and thermal sensitivity and induced place aversion.

### 3.4 The mutual projections between the RSC and MCC

**Figure 4:**
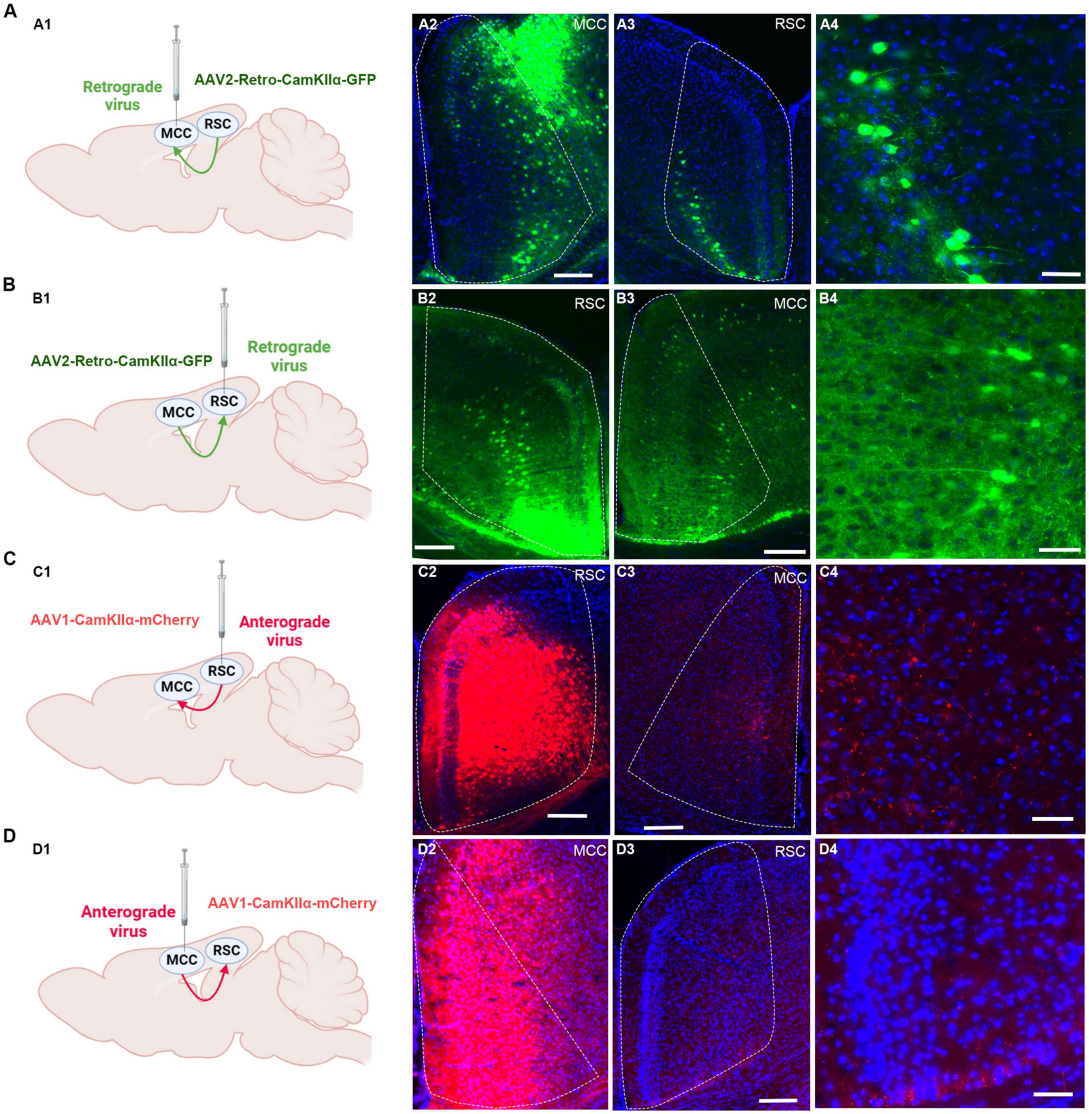
The mutual projections between the RSC and MCC. **(A)** The projections from RSC to MCC were evaluated via retrograde virus. **(A1)** Scheme for specific labeling of MCC neurons projecting to RSC (created on BioRender.com). **(A2)** The injection site of the virus in the MCC. **(A3)** The RSC neurons expressing EGFP. **(A4)** An enlarged image showing the EGFP expressing neurons in RSC from **A2**. **(B)** The projections from MCC to RSC were evaluated via retrograde virus (created on BioRender.com). **(B1)** Scheme for specific labeling of RSC neurons projecting to MCC. **(B2)** The injection site of the virus in the RSC. **(B3)** The MCC neurons expressing EGFP. **(B4)** An enlarged image showing the EGFP-expressing neurons in MCC from **B3**. **(C)** The projections from RSC to MCC were evaluated via anterograde virus (created on BioRender.com). **(C1)** Scheme for specific labeling of RSC neurons projecting to MCC. **(C2)** The injection site of the virus in the RSC. **(C3)** The mCherry-expressing fibers in the MCC. **(C4)** An enlarged image showing the mCherry expressing fibers in MCC from **B3**. **(D)** The projections from MCC to RSC were evaluated via anterograde virus (created on BioRender.com). **(D1)** Scheme for specific labeling of MCC neurons projecting to RSC. **(D2)** The virus injection site in the MCC. **(D3)** the mCherry-expressing fibers in the RSC. (D4) The enlarged image showing the mCherry expressing fibers in RSC from **D3**.

We proceeded to investigate the potential projections between the RSC and MCC (Figure 4A). Initially, we administered the rAAV2/1-CaMKIIα-mCherry virus, an anterior-transneuronal virus, into the RSC. Following stable expression, we observed the expression of GFP in the soma of MCC neurons, indicating that neurons from the RSC project to the MCC (Figure 4B). Employing a similar method, we injected rAAV2/1-CaMKIIα-mCherry into the MCC and observed mCherry-positive cells in the RSC (Figure 4C). To further validate the mutual projection between these two brain regions, we administered the rAAV2-retro-GFP, a retrograde virus, into the RSC or MCC, respectively. Correspondingly, we detected GFP-positive cells in the MCC and RSC, confirming the reciprocal projections between the two areas (Figures 4D,E). These tracing results strongly indicate the existence of mutual projections between the RSC and MCC.

**Figure 5:**
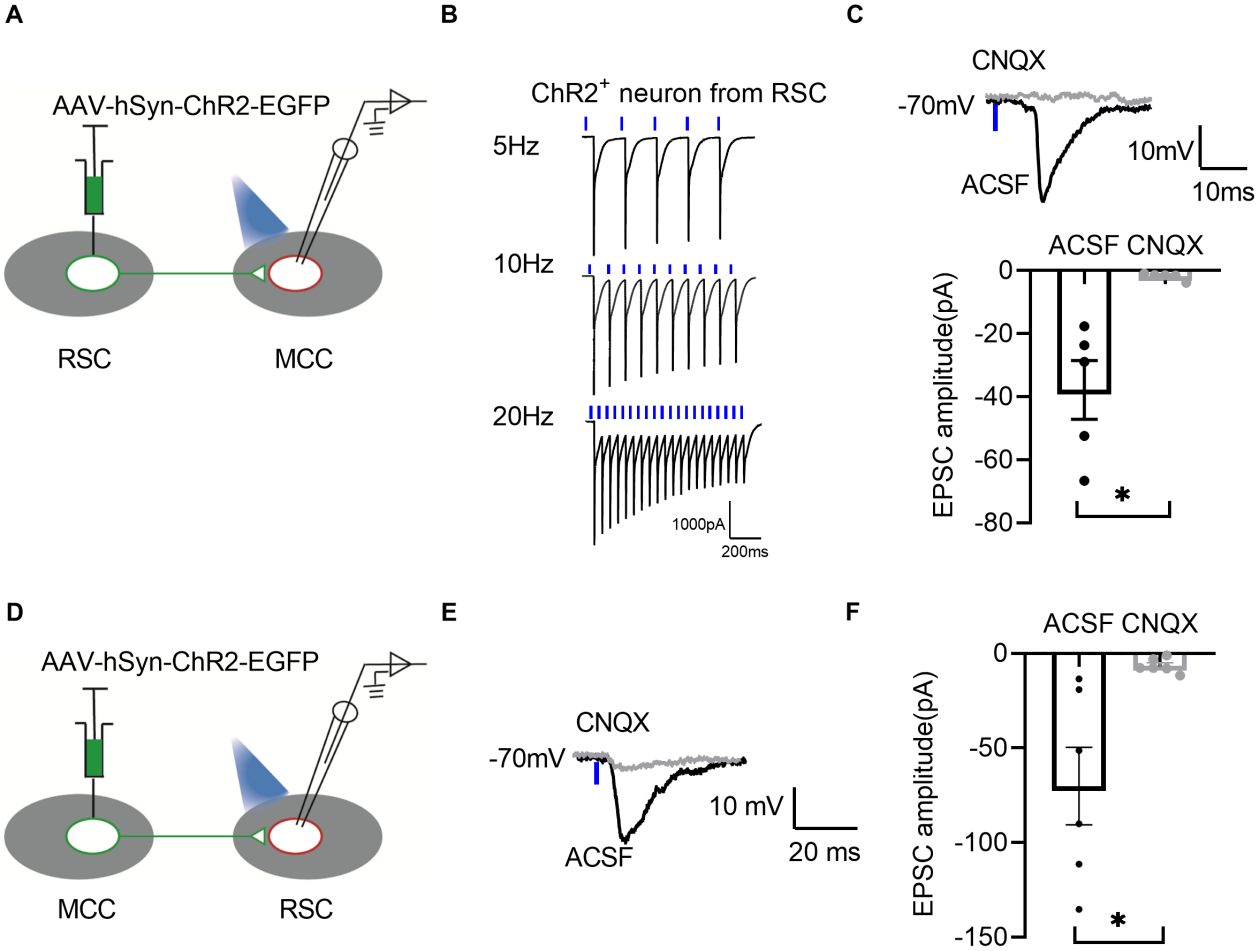
There are bidirectional excitatory synaptic connections between the RSC and MCC. **(A)** Schematic showing MCC electrophysiological recordings in acute slices. **(B)** Representative traces showing activation of the ChR2-GFP-expressing neuron by 1 ms light stimulation. **(C)** Up: Representative traces showing light-evoked EPSC from MCC neurons. Bottom: Summary data for the amplitudes of light-evoked EPSC from MCC neurons (paired *t*-test, two-tailed, *t* = 3.829, df = 4, *p* = 0.0186, 5 neurons from 3 mice). **(D)** Schematic showing RSC electrophysiological recordings in acute slices. **(E)** Representative traces showing light-evoked EPSC from RSC neurons. **(F)** Summary data for the amplitudes of light-evoked EPSC from RSC neurons (paired *t*-test, two-tailed, *t* = 3.290, df = 5, *p* = 0.0217, 6 neurons from 3 mice).

To further confirm the synaptic connections between RSC and MCC, we performed the whole-cell patch-clamp recording experiments. We first injected the AAV-hsyn-ChR2-EGFP in the RSC (Figure 5A), and the neurons in the RSC responded well to the blue laser stimulations at 5, 10, and 20 Hz (Figure 5B), we then examined the possible synaptic transmissions on the MCC neurons with the blue laser stimulations. Interestingly, we recorded the inward currents under the voltage-clamp model, which were blocked by the application of 6-cyano-7-Nitroquinoxaline-2,3-dione (CNQX, 20 μM), an antagonist of the AMPA/Kainate receptors (Figure 5C), indicating the existing of the AMPARs/Kainate receptors mediating synaptic transmissions from the RSC to the MCC. We then injected the AAV-hsyn-ChR2-EGFP into the MCC (Figure 5D), and similarly, we recorded the synaptic responses on the RSC neurons under blue laser stimulations (Figures 5E,F). Therefore, the electrophysiological recording results confirmed the mutual connections between the RSC and MCC.

### 3.5 The MCC is necessary to the RSC’s regulation of pain

**Figure 6:**
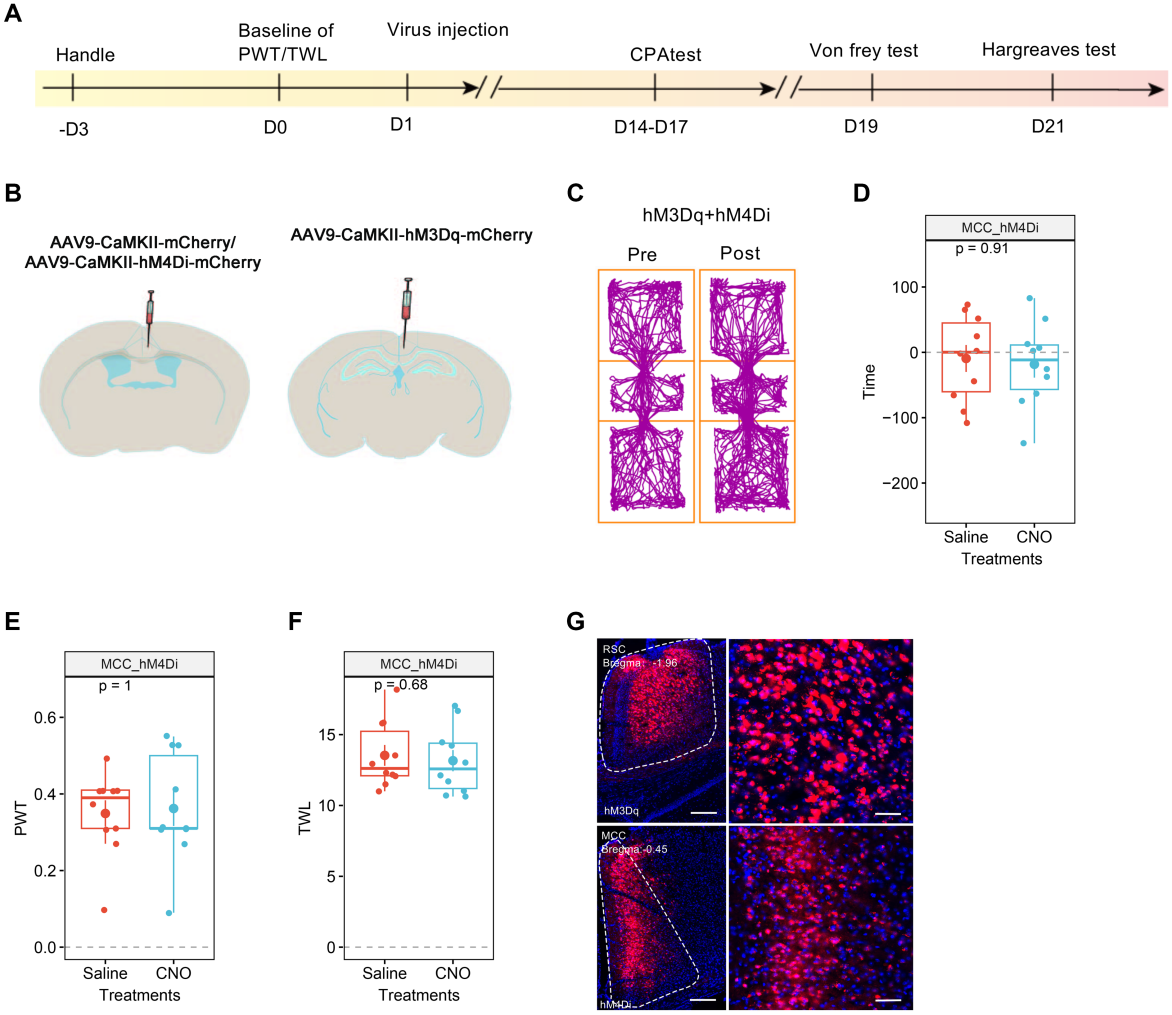
The neurons in MCC gated the regulations of RSC neurons on PWT and TWL. **(A)** The cartoon diagram illustrates the experimental procedure of applying chemogenetics to elevate neuronal activity in the RSC but inhibits the neuronal activity in MCC and to perform behavioral assessments in the current research. **(B)** Schematic diagram of virus injection in the MCC and RSC. **(C)** Representative traveling traces of the Ctrl/hM4Di (MCC) group of mice in the conditioned place aversion test, the hM3Dq were also expressed in RSC. **(D)** The CNO application in the hM3Dq (RSC) / hM4Di (MCC) group did not induce place aversion compared to the hM3Dq (RSC)/Ctrl (MCC) in Figure 2D. (Two-way RM ANOVA, Interaction, *F*_(1, 24)_ = 7.01, *p* < 0.05, Saline vs. CNO: *F*_(1, 24)_ = 21.90, *p* < 0.0001, Ctrl vs. hM4Di: *F*_(1, 24)_ = 3.34, *p* = 0.08; Tukey’s multiple comparisons test, hM4Di: Saline vs. CNO, *p* = 0.27, Ctrl: *n* = 12, hM4Di: *n* = 14). **(E)** The application of CNO has no effect on the PWT in hM3Dq (RSC)/hM4Di (MCC)-expressing mice compared to the hM3Dq (RSC)/Ctrl (MCC) in Figure 2E. (Two-way ANOVA, Interaction, *F*_(1, 20)_ = 51.08, *p* < 0.0001, Saline vs. CNO: *F*_(1, 20)_ = 45.63, *p* < 0.0001, Ctrl vs. hM4Di: *F*_(1, 20)_ = 6.80, *p* < 0.05; Tukey’s multiple comparisons test, hM4Di: Saline vs. CNO, *p* = 0.96, Ctrl: n = 12, hM4Di: *n* = 10). **(F)** The application of CNO did not change TWL in hM3Dq (RSC) /hM4Di (MCC)-expressing mice compared to the hM3Dq (RSC)/Ctrl (MCC) in Figure 2F. (Two-way ANOVA, *F*_(1, 20)_ = 74.48, *p* < 0.0001, Saline vs. CNO: *F*_(1, 20)_ = 90.55, *p* < 0.0001, Ctrl vs. hM3Dq: *F*_(1, 20)_ = 11.05, *p* < 0.05; Tukey’s multiple comparisons test, hM4Di: Saline vs. CNO, *p* = 0.80, Ctrl: *n* = 12, hM4Di: *n* = 10). **(G)** Representative images showing expression of control or hM3Dq-mCherry in the MCC.

Given that both RSC and MCC regulate the pain sensation, we sought to determine the necessity of the MCC or RSC when the other region was activated. To address this question, we injected AAV9-CaMKIIα-hM3Dq-mCherry into the RSC and AAV9-CaMKIIα-hM4Di-mCherry into the MCC. Subsequently, systemic application of CNO would activate RSC neurons while inhibiting the MCC neurons (Figures 6A,B). Intriguingly, CNO failed to induce place aversion in mice with hM3Dq (RSC) and hM4Di (MCC) expression (Figures 6C,D). Furthermore, in freely behaving animals, CNO application did not decrease the PWT (Figure 6E) or shorten the TWL in mice expressing hM4Di (Figure 6F). The expression of the viruses was confirmed through imaging (Figure 6G). These findings strongly suggest that the activation of MCC neurons is necessary to regulate pain via the RSC.

### 3.6 The RSC is critical to the MCC’s regulation of thermal sensation

**Figure 7:**
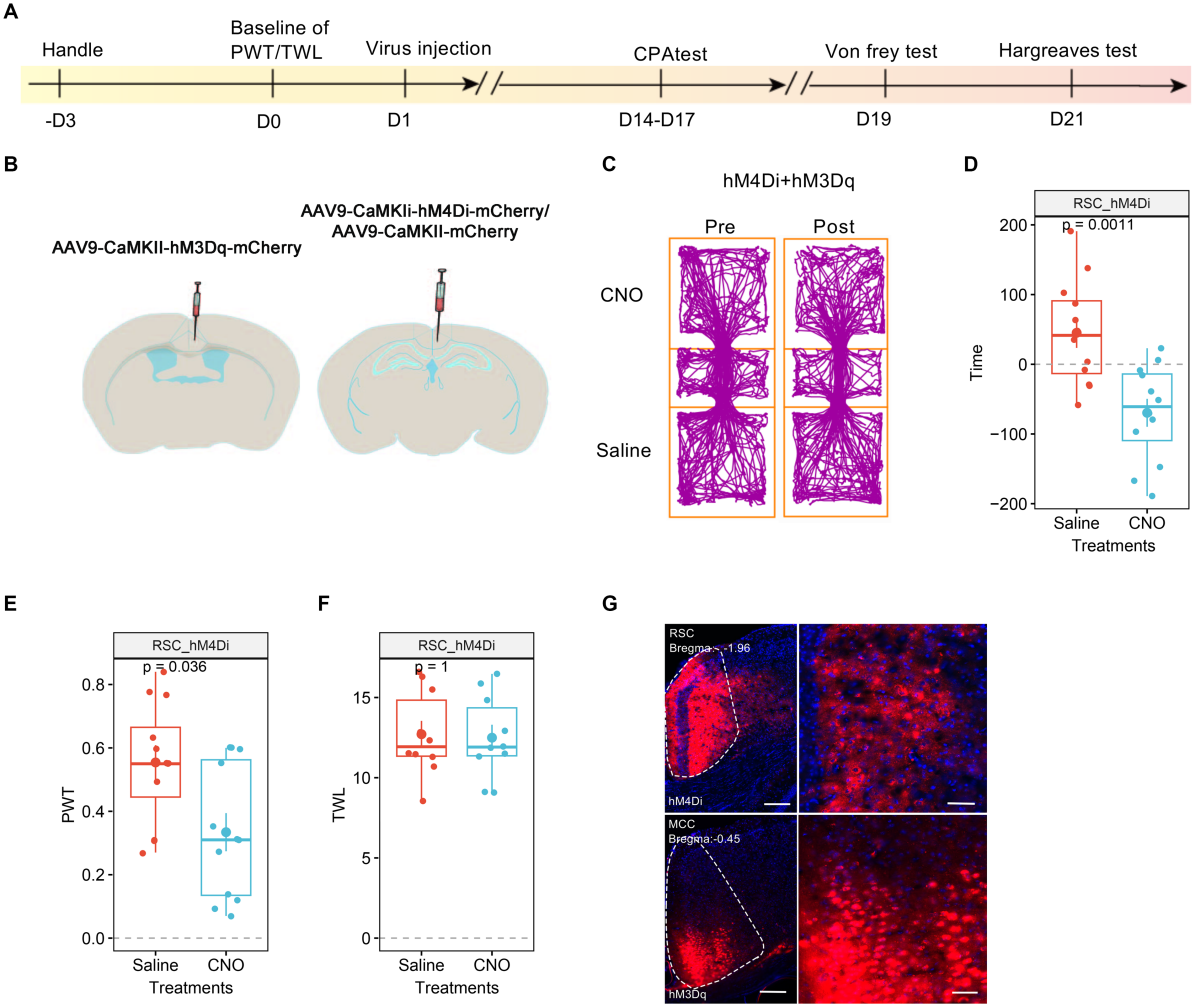
The neurons in RSC were necessary to the MCC’s regulations on thermal response. **(A)** The cartoon diagram illustrates the experimental procedure of applying chemogenetics to elevate neuronal activity in the MCC but inhibits the neuronal activity in RSC and to perform behavioral assessments in the current research. **(B)** Schematic diagram of virus injection in the MCC and RSC. **(C)** Representative traveling traces of the mice with Ctrl/hM4Di in RSC and hM3Dq in MCC in the conditioned place aversion test. **(D)** The CNO application in the hM4Di (RSC)/hM3Dq (MCC) group did induce place aversion (compared to the Ctrl (RSC)/hM3Dq (MCC) group in Figure 3D. Two-way ANOVA, Interaction, *F*_(1, 24)_ = 0.07, *p* = 0.79, Saline vs. CNO: *F*_(1, 24)_ = 24.86, *p* < 0.0001, Ctrl vs. hM4Di: *F*_(1, 24)_ = 2.54, *p* = 0.12; Tukey’s multiple comparisons test, hM4Di: Saline vs. CNO, *p* < 0.01, Ctrl: *n* = 14, hM4Di: *n* = 12). **(E)** The application of CNO decreased the PWT in hM3Dq-expressing mice (compared to the Ctrl (RSC)/hM3Dq (MCC) group in Figure 3E, two-way ANOVA, Interaction, *F*_(1, 23)_ = 0.0002, *p* = 0.99, Saline vs. CNO: *F*_(1, 23)_ = 14.84, *p* < 0.001, Ctrl vs. hM4Di: *F*_(1, 23)_ = 17.08, *p* < 0.001; Tukey’s multiple comparisons test, hM4Di: Saline vs. CNO, *p* < 0.05, Ctrl: *n* = 13, hM4Di: *n* = 12). **(F)** The application of CNO extended the TWL in hM3Dq-expressing mice (compared to the Ctrl (RSC)/hM3Dq (MCC) group in Figure 3F, two-way ANOVA, Tukey’s multiple comparisons test, Interaction, *F*_(1, 19)_ = 13.38, *p* < 0.01, Saline vs. CNO: *F*_(1, 19)_ = 15.43, *p* < 0.001, Ctrl vs. hM4Di: *F*_(1, 19)_ = 9.68, *p* < 0.01; Tukey’s multiple comparisons test, hM4Di: Saline vs. CNO, *p* = 0.98, Ctrl: *n* = 11, hM4Di: *n* = 10). **(G)** Representative images showing expression of control or hM3Dq-mCherry in the MCC.

We proceeded to investigate the roles of RSC in the pain regulation of the MCC. For this purpose, we injected AAV9-CaMKIIα-hM3Dq-mCherry into the MCC and AAV9-CaMKIIα-hM4Di-mCherry into the RSC (Figures 7A,B). Similar to our previous findings, the administration of CNO did not induce significant place aversion (Figures 7C,D). In freely behaving animals, CNO application still decreased the PWT in mice expressing hM4Di-mCherry in the RSC (Figure 7E). However, it failed to shorten the TWL (Figure 7F), and Figure 7G depicted the expression of the viruses in the MCC and RSC. Taken together, these observations suggest that the activation of RSC neurons is necessary for regulating the MCC’s response to thermal stimulation.

## 4 Discussion

In this study, we assessed the roles of the MCC and RSC in pain regulation using a chemogenetic approach. Our findings demonstrated that chemogenetically elevating the activities of neurons in either the RSC or MCC heightened pain sensitivity. Interestingly, inhibiting the MCC eliminated the RSC’s ability to regulate pain. Additionally, our results revealed that the RSC plays a crucial role in the MCC’s regulation of thermal sensation. These results highlight the distinct roles of the RSC and MCC in pain modulation.

### 4.1 The involvement of RSC and MCC in pain regulation

The involvement of MCC in pain regulation has been studied extensively. Previous studies have demonstrated that painful laser stimulation evoked responses in the poster midcingulate cortex in humans (Frot et al., 2008). Furthermore, it has been reported that MCC neurons responded to noxious stimulations (Sikes et al., 2008). Optogenetic approaches have been employed to manipulate MCC neural activities bilaterally, revealing that MCC regulates sensory hypersensitivity across a wide cortical and subcortical area. Interestingly, activating glutamatergic neurons in area 2 (Cg2^Glu^) of MCC using a blue laser in mice receiving the ChR2 virus led to a significant increase in PWT and TWL; conversely, optogenetically activated MCC Cg1^Glu^ exhibited an opposing function in pan modulation (Hu et al., 2019). Here, we chemogenetically activated the RSC and the entire MCC to investigate their involvement in regulating pain. We found that for the mice expression AAV9-CaMKIIα-hM3Dq-mCherry in RSC or MCC, the application of CNO, which will elevate the activities of virus-expressing neurons, decreased the PWT and TWL. Consistently, in Tan’s et al. (2017) study, they optogenetically increased the activities of MCC neurons in awake rAAV-CamkIIα-ChR2-expressing mice and observed a marked hypersensitivity. Therefore, both studies indicated that the higher level of MCC’s neuronal activities sensitized the pain sensation. In Tan’s et al. (2017) study, they showed that the optogenetically activated MCC neurons did not induce CPA. Here we observed the difference between the saline and CNO paired chambers in the mice with AAV9-CaMKIIα-hM3Dq-mCherry in the MCC, which indicates the involvements of the MCC to the negative effects in the CPA. We believe that the primary distinction arises from the methods of activation. In Tan’s et al. (2017) study, the manipulations lasted for 15 min, while for the chemogentic approach, the manipulations may lasted for a longer time, which may induce further effects on negative effects on the CPA, and further studies are needed to evaluate the role of MCC in the negative emotion.

### 4.2 The interaction between RSC and MCC contributes to pain regulation

In the current study, we observed the mutual projections between the RSC and MCC by virus tracing approaches, and we also detected the functional synaptic transmission between two brain regions by whole-cell path-clamp recording approach, interestingly, most of the evoked synaptic responses were blocked by the bath application of CNQX, an antagonist of AMPAR/kainite receptors, these results indicated that the glutamate-mediated synaptic transmissions between two brain regions. Further studies should be performed to examine the existence of inhibitory synaptic transmissions between two brain areas.

We utilized a chemogenetic approach to modulate the functions of MCC and RSC and examined their distinct roles in pain regulation. Our findings revealed that activating the MCC or RSC alone heightened sensitivity to both mechanical and thermal stimuli. However, only mechanical sensation was sensitized when the MCC was activated alongside RSC inhibition, while thermal sensation remained unaffected. This suggests that RSC neuronal activity plays a crucial role in the MCC’s regulation of thermal sensitivity. In line with our previous study (Wang et al., 2023), elevating the neuronal activities in the RSC alone decreased the PWT, shortened the TWL, and induced place aversion, while in the mice with the expression of hM4Di in MCC and hM3Dq in the RSC, the application of CNO would inhibit the MCC’s neuronal activities and enhance the RSC’s neuronal activities, on the behavioral level, the CNO did not affect the PWT, TWL and induce place aversion, these results indicate the critical role of MCC in RSC’s regulation on pain sensation. The differential effects on mechanical and thermal sensation when modulating the MCC and RSC suggest a complex interplay between these regions in pain processing. The varying effects could arise from the distinct circuits within MCC and RSC. As observed in previous studies, the MCC modulates the motor-related functions of the cingulate cortex due to its projection to the spinal cord (Vogt and Vogt, 2003; Hoffstaedter et al., 2014; Misra and Coombes, 2015). It has also been demonstrated that anticipated pain triggers increased activation in the MCC, which directly correlates with motor output (Koppel et al., 2023). Our findings suggest that the inhibition of MCC may impede the transmission of pain signals to motor centers, potentially explaining the absence of pain-related behaviors despite chemogenetic manipulation. This highlights the complexity of pain processing, where sensory input and motor output are intricately linked.

## 5 Conclusion

Our findings, in conjunction with prior research, highlight the MCC and RSC as key players in pain regulation and aversion. The chemogenetic modulation of these regions provides a nuanced understanding of their distinct roles and interactions in pain processing. Future studies should focus on elucidating the underlying circuitry and the temporal dynamics of MCC-RSC interactions to fully appreciate their contributions to pain sensation and aversion.

## Data availability statement

The raw data supporting the conclusions of this article willbe made available by the authors, without undue reservation.

## Ethics statement

The animal study was approved by The Zhejiang University Animal Care and Use Committee approved all mouse protocols. The study was conducted in accordance with the local legislation and institutional requirements.

## Author contributions

YQ: Investigation, Writing - review & editing. Y-NL: Investigation, Writing – original draft. CW: Writing – review & editing. LL: Methodology, Writing – review & editing. CZ: Conceptualization, Writing – original draft. X-YL: Conceptualization, Writing – original draft, Writing – review & editing.
